# Zinc oxide nanostructures for fluorescence and Raman signal enhancement: a review

**DOI:** 10.3762/bjnano.13.40

**Published:** 2022-05-27

**Authors:** Ioana Marica, Fran Nekvapil, Maria Ștefan, Cosmin Farcău, Alexandra Falamaș

**Affiliations:** 1 National Institute for Research and Development of Isotopic and Molecular Technologies, 67-103 Donat, 400293 Cluj-Napoca, Romania; 2 Biomolecular Physics Department, Babeș-Bolyai University, 1 Kogălniceanu, 400084 Cluj-Napoca, Romania; 3 RDI Laboratory of Applied Raman Spectroscopy, RDI Institute of Applied Natural Sciences (IRDI-ANS), Babeş-Bolyai University, Fântânele 42, 400293, Cluj-Napoca, Romania

**Keywords:** fluorescence, surface-enhanced Raman spectroscopy, ZnO–metal nanomaterials, ZnO nanostructures

## Abstract

Since the initial discovery of surface-enhanced Raman scattering (SERS) and surface-enhanced fluorescence (SEF), these techniques have shown huge potential for applications in biomedicine, biotechnology, and optical sensors. Both methods rely on the high electromagnetic fields created at locations on the surface of plasmonic metal nanoparticles, depending on the geometry of the nanoparticles, their surface features, and the specific location of analyte molecules. Lately, ZnO-based nanostructures have been exploited especially as SERS substrates showing high enhancement factors and increased charge transfer effect. Additionally, applications focused on enhancing the fluorescence of analyte molecules as well as on tuning the photoluminescence properties of ZnO nanostructures through combination with metal nanoparticles. This review covers the major recent results of ZnO-based nanostructures used for fluorescence and Raman signal enhancement. The broad range of ZnO and ZnO–metal nanostructures synthesis methods are discussed, highlighting low-cost methods and the recyclability of ZnO-based nanosubstrates. Also, the SERS signal enhancement by ZnO-based nanostructures and the influences of lattice defects on the SERS signal are described. The photoluminescence enhancement of ZnO in the presence of noble metal nanoparticles and the molecular fluorescence enhancement in the presence of ZnO alone and in combination with metal nanoparticles are also reviewed.

## Introduction

Over the last decades, ZnO-based nanomaterials have been extensively used in the industry and investigated in various application fields such as optoelectronics, biomedicine, agriculture, food, and cosmetics [1–3]. The wide range of applications is due to the many promising features of ZnO nanoparticles (NPs), such as their wide bandgap energy (3.3–3.7 eV), strong luminescence [4–5], antibacterial properties, and UV-protection properties. Additionally, ZnO nanomaterials can be designed into various morphologies, such as nanoparticles, nanoneedles, nanorods, nanocages, nanocombs, and nanoflowers [5–8].

Hybrid nanomaterials can be obtained by combining ZnO with metal NPs, thus integrating the material properties of both components and resulting in new and enhanced properties that are not obtainable from the single component nanoparticles. Recent studies showed that ZnO properties can be tuned and improved when combined with metal nanoparticles, resulting in enhanced photoactivity of Au-decorated ZnO nanocrystals for photoelectrochemical water splitting [9], improved photodetection performance of ZnO nanofibers decorated with Au NPs [10], or enhanced photocatalytic activity of ZnO doped with Au NPs [11]. Moreover, enhanced Raman scattering for periodic ZnO-elevated Au dimer nanostructures [12] and enhanced fluorescence emission signals from Al-doped ZnO films [13] were obtained. The development of hybrid nanocomposites based on ZnO and noble metals for fluorescence and Raman signal enhancement has recently attracted great interest and will be the focus of this review.

The electromagnetic (EM) enhancement in surface-enhanced Raman scattering (SERS) appears due to the enhanced local electric field that is generated when localized surface plasmon resonances (LSPRs) are excited by light incident on noble metal nanostructures. Besides this field enhancement, the excitation of the collective oscillations of the conduction electrons in metallic NPs results in strong absorption and scattering of light in the visible range of the electromagnetic spectrum. Moreover, even larger EM field enhancement can be obtained in noble metal NPs through the creation of “hot spots”, small gaps between NPs (less than 10 nm), due to interparticle EM coupling under the incident light. Similarly, large field enhancements can arise around sharp tips and edges on the rough surface of NPs. The EM enhancement effects on semiconductor nanostructures, however, are significantly inferior to those on noble metals since the LSPR is centred in the near-infrared in the case of the conduction band (CB) and in the UV region in the case of the valence band (VB) [14]. Therefore, concrete solutions have been proposed to improve the EM enhancement in ZnO nanostructures by combining them with noble metal NPs and tuning the peak location of the LSPR near to the visible region of the EM spectrum and by creating “hot spots” in ZnO ordered nanostructures [15]. This results in strong scattering and absorption of incident light, thus influencing optical processes such as SEF, SERS [16–17], infrared absorption, and even second harmonic generation [18], which can improve the performance of optical sensors and optoelectronic devices. ZnO alone and in combination with noble metals has been recently used for the development of SERS substrates [15,19] due to several properties including a high refractive index, which can confine the excitation light in order to enhance the SERS effect, various types of tuneable morphologies that can be used in combination with noble metals, but also its biocompatibility, photocatalytic self-cleaning capability, and high chemical stability, to name just a few. Additionally, ZnO-based nanosubstrates have been used lately in combination with noble metals to enhance the fluorescence emission of dyes located in their vicinity [20–21] as well for amplifying the fluorescence emission of ZnO, which could prove very useful for solar cell applications [13].

This review article seeks to present the fabrication methods and applications of zinc oxide nanostructures enhancing the photoluminescence emission or the Raman signal of analyte molecules. First, we focus on a wide range of synthesis methods of ZnO nanostructures and nanocomposites based on ZnO and noble metals. Second, the Raman enhancing capabilities and advantages of ZnO-based nanostructures as SERS substrates are discussed. Last, we focus on methods for enhancement of ZnO photoluminescence by noble metal nanoparticles and the enhancement of molecular fluorescence by ZnO alone and by ZnO–metal hybrid nanoparticles. Moreover, we consider the physical phenomena governing both the Raman and fluorescence enhancement using ZnO nanostructures.

## Review

### Fabrication of ZnO–noble metal nanocomposites

#### Synthesis of ZnO nanostructures

Among the exhaustive list of available physical and chemical methods used for obtaining ZnO nanoparticles, solution synthesis methods such as sol–gel [22], chemical precipitation [23], polyol [24], and solvothermal [25] methods, are inexpensive, consume little energy, allow for a facile control of physical characteristics and morphology of the nanoparticles, offer good reproducibility, and are usually effective for large scale production. The chemical precipitation technique has gained increased attention due to its simplicity and efficiency for obtaining nanosized ZnO structures with various morphologies, such as nanoparticles, nanorods, nanoneedles, and nanotetrapods [26]. Size and shape of the ZnO NPs depend on the type of precursor salts used (e.g., chlorides, sulfates, nitrates, or perchlorates), the molar ratio between reagents, the reaction temperature, the pH value, the ionic concentration of the medium, and other reaction parameters. The main advantage of this synthesis method is that through the co-precipitation reaction a large amount of nanoparticles can be easily obtained. Moreover, the chemical precipitation method allows for rigorous control over nuclear and particle growth in solution.

The polyol method offers good control of the shape and size of the synthesized products, accompanied by a uniform granulomere distribution, relatively low reaction temperature, and a reaction time of the order of minutes. Also, the used polyols are environmentally harmless and biocompatible. The method has several particularities due to the involved polyols, such as high boiling point (up to 320 °C) and dielectric constant, the solubility of simple metal salt precursors, and coordinating properties for surface functionalization preventing agglomeration [27]. The ZnO NPs obtained from polyol synthesis showed excellent crystalline quality and controllable morphology [28].

Various morphologies including nanoworms, nanowires, and nanorods with excellent crystallinity were also obtained using the solvothermal method [25]. According to Chieng et al., the particle sizes of the synthesized ZnO nanoparticles are in correlation with the glycol chain length. With increasing the glycol chain length, the average particle size of ZnO is also increased [27]. Employing ethylene glycol as a polyol led to assembled hollow sphere structures, while diethylene glycol resulted in elliptical nanorod structures [29].

**Figure 1:**
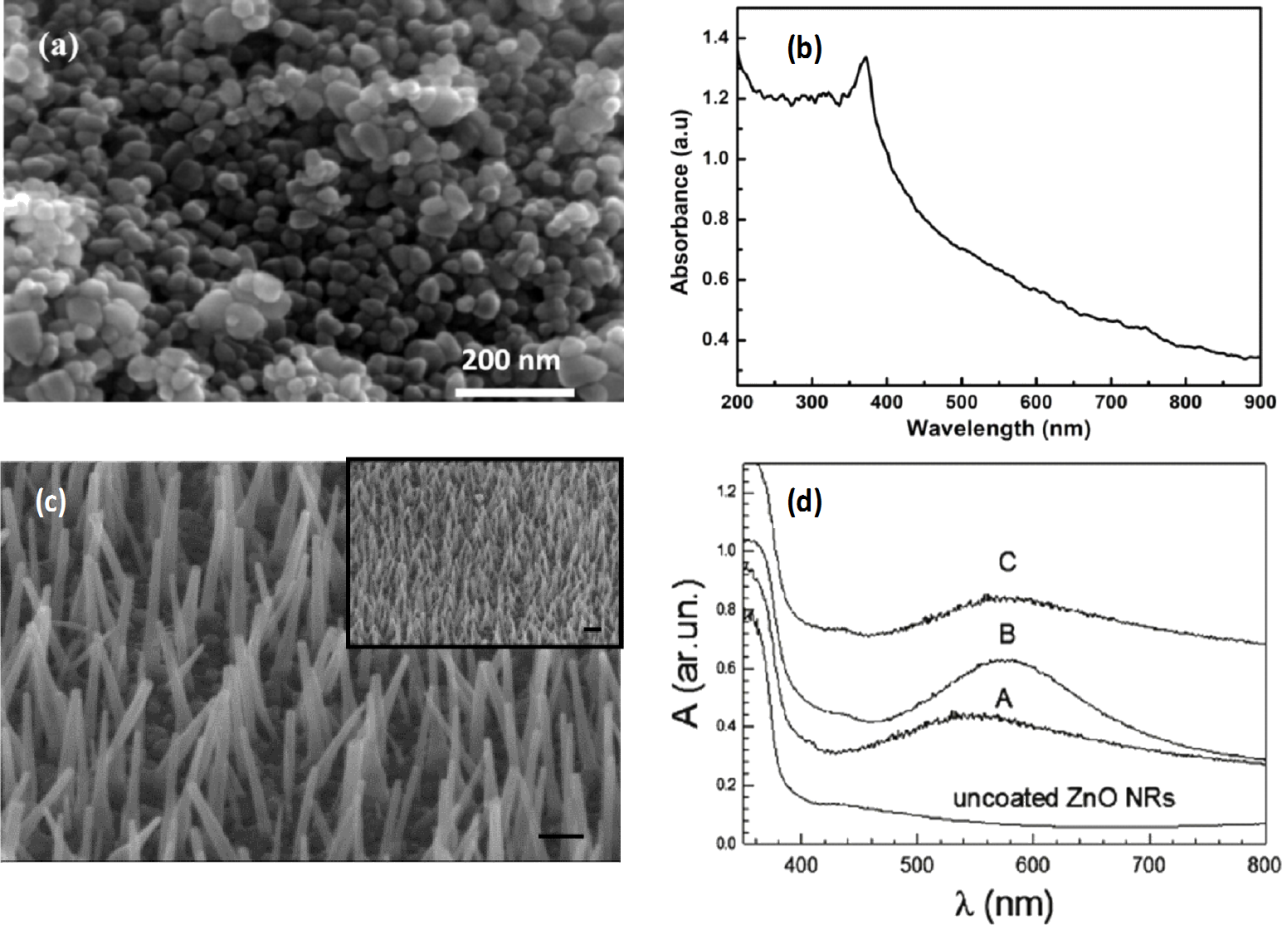
SEM of hydrothermally grown ZnO nanoparticles (a) and of vertically aligned ZnO nanords (c). UV–vis absorption spectra of spherical ZnO nanoparticles (b) and of ZnO nanorods coated with Au at different sputtering time: 20 s (sample A), 30 s (sample B), and 60 s (sample C) (d). Figure 1 (a) and (b) were reproduced from [8] (B. P. Majee et al., “Bi-functional ZnO nanoparticles as a reusable SERS substrate for nano-molar detection of organic pollutants”, Mater. Res. Express, vol. 6, article no. 1250j1, published on 7 February 2020; https://doi.org/10.1088/2053-1591/ab6f31); © 2020 IOP Publishing Ltd. Reproduced with permission via Copyright Clearance Center. All rights reserved. This content is not subject to CC BY 4.0. Figure 1 (c) and (d) were adapted with permission from [6]. Copyright (2011) American Chemical Society. This content is not subject to CC BY 4.0.

The simplicity and environmentally friendly conditions involved in the hydrothermal synthesis of ZnO make it an attractive growth method, widely used in recent years. A comprehensive review indicating the morphology of ZnO nanostructures grown using this method is given in [30]. Successful examples of ordered ZnO nanorods [6], one of most common ZnO shapes used for SERS applications, as well as spherical ZnO nanoparticles [8] are given in Figure 1.

#### Combination of ZnO nanostructures with noble metals

The use of inexpensive semiconducting materials, such as wide-bandgap ZnO or TiO_2_, for SPR-based substrates has gained increased attention in recent years due to important advantages that they offer, for example, good recyclability, long-term use, and cost effectiveness. For ultrasensitive detection of molecules, however, the SERS performance of standalone semiconductor substrates is too weak. Therefore, the development of hybrid nanomaterials based on semiconductors decorated with noble metals, or vice versa, has been proposed. These hybrid nanostructures show enhanced optical and electronic properties due to the coupling between the noble metal and the semiconductor [10–11,13].

**Figure 2:**
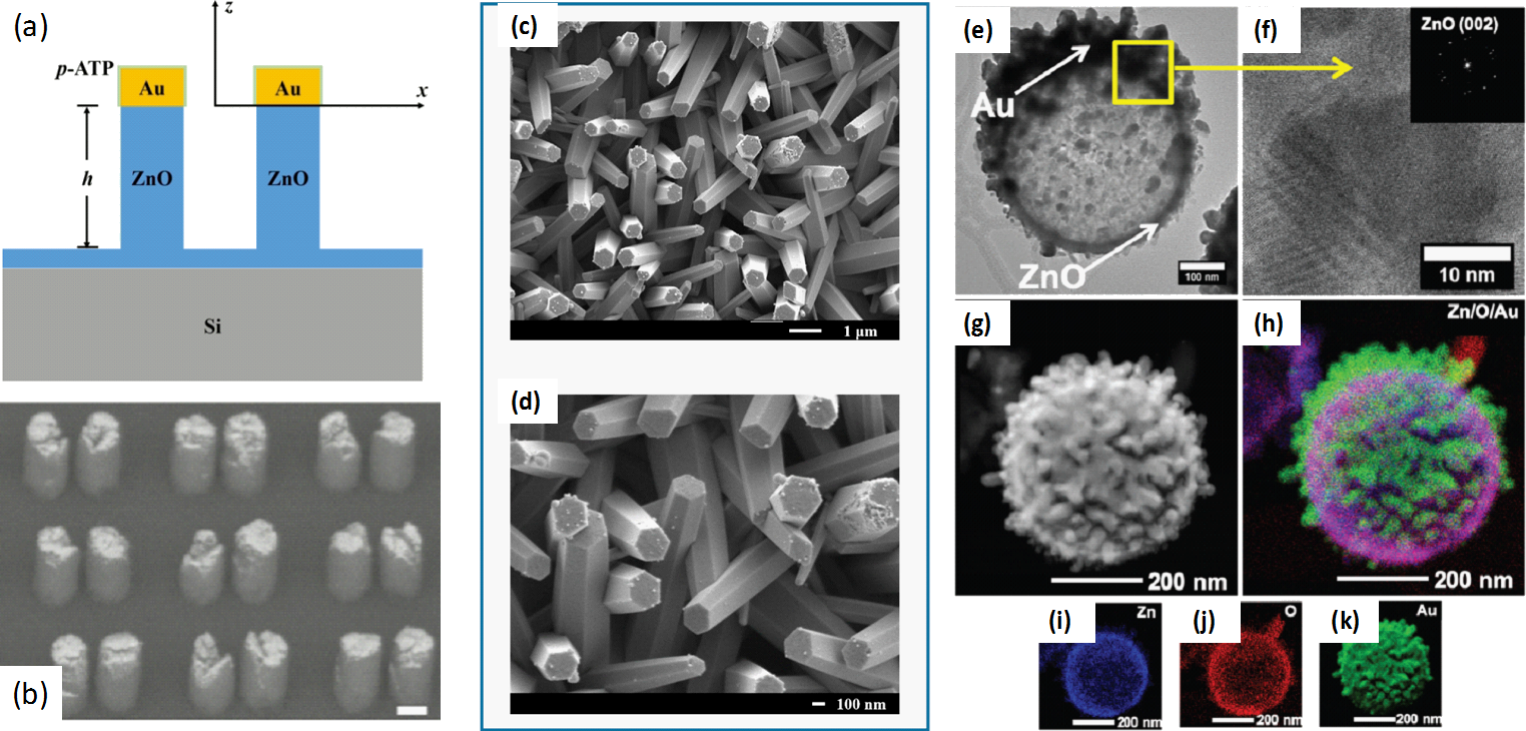
Schematic illustration (a) and SEM (b) of ZnO nanorods obtained by electron-beam lithography and hydrothermal growth with Au nanostructures deposited on their surface. The SEM illustrates ZnO nanorods of 200 nm height, 75 nm Au dimer size and 30 nm gap sizes between the nanostructures. Adapted with permission from [12] Copyright (2018) American Chemical Society. This content is not subject to CC BY 4.0. SEM images (c and d) of Au-decorated ZnO nanorods, where the ZnO nanorods were fabricated using unannealed seed layers and the Au NPs were deposited by pulsed-laser-induced photolysis. Figure 2 (c) and (d) were adapted from [38] (© 2020 Chou, C. M. et al., published by MDPI, distributed under the terms of the Creative Commons Attribution 4.0 International License, https://creativecommons.org/licenses/by/4.0). TEM image (e) of ZnO-Au nanostructures and the high-resolution image (f) of the selected area in (e). STEM (g) and EDX elemental mapping images displaying the ZnO-Au structure (h), Zn (i) and O (j) location in the central part of the nanostructure, respectively Au (k) on its surface. Figure 2 e-k was republished (adapted) with permission of The Royal Society of Chemistry from [16] (“Au-covered hollow urchin-like ZnO nanostructures for surface-enhanced Raman scattering sensing” by O. Graniel et al., J. Mater. Chem. C, vol. 7, issue 47, © The Royal Society of Chemistry 2019; https://doi.org/10.1039/C9TC05929F; permission conveyed through Copyright Clearing Center, Inc.). This content is not subject to CC BY 4.0.

Various fabrication techniques have been employed to develop ordered hybrid nanostructured substrates, ranging from more expensive and laborious ones, such as pulsed laser deposition or hydrothermal growth, followed by sputtering processes [31] or electron beam lithography to more cost-efficient and simple ones, such as photochemical deposition of metallic NPs or a metallic layer [32], chemical synthesis [33], or nanosphere lithography. Usually, ZnO nanostructures are fabricated first, followed by the decoration with metallic nanostructures or a metallic layer, which is added by physical vapour deposition, including sputtering processes [6,34], ion sputtering, which allowed for the decoration of vertically aligned cone-shaped ZnO nanorods with Ag NPs on their sides and their top ends [35], and magnetron sputtering [7,36]. Electron beam evaporation of, for example, a 30 nm Au layer on ZnO nanopillars arrays [37] or of ZnO-elevated Au dimer nanostructures (Figure 2a,b) [12] was carried out as well. Chou et al. employed a simple and rapid method, namely pulsed laser-induced photolysis to develop Au NPs on the surface of ZnO nanorods fabricated by the sol–gel method (Figure 2c,d) [38]. Various irradiation times were tested, indicating that a short irradiation time was needed to develop efficient SERS substrates. The combination of several methods including nanosphere lithography, atomic layer deposition, electrodeposition, and electron-beam evaporation resulted in Au-covered hollow urchin-like ZnO structures (Figure 2e–k) [16]. The ZnO layer was deposited on a substrate covered with polystyrene spheres by atomic layer deposition, followed by electrodeposition, which was used to grow ZnO nanowires onto the surface. The Au layer was deposited after burning off the polystyrene spheres by electron beam evaporation while monitoring its thickness.

Considering the growing demand for metal–semiconductor-based nanocomposites for industrial and biomedical applications, there is a critical need to develop more facile and versatile synthetic routes for obtaining these nanocomposites [39]. Traditional chemical synthesis methods used for developing semiconductor–noble metal-based nanocomposites can present several disadvantages, such as the use of complex polymer stabilizers, which result in toxic activity [40], SERS deactivation [41], or undesired signals in SERS analysis. Therefore, simple and rapid metal decoration methods have been applied recently. Some of these include hydrothermal method [42], controlled wet chemistry using spin coating, which can deposit, for example, uniform Ag NPs onto ZnO nanowhiskers and nanocolumns [43], photochemical deposition, which allows the formation of different Au shapes depending on the type of additive added into the photochemical reduction [32], pulsed laser-induced photolysis [38], or controlled decoration with Ag NPs using an electroless plating technique [44]. Photochemical synthesis permits the control of nucleation and growth rate without using organic additives. Xu et al. employed laser irradiation of ZnO nanorods in HAuCl_4_ to controllably grow Au NPs on the ZnO nanorods and obtained effective substrates for the ultrasensitive detection of organic pollutants in water, which could be recycled multiple times [45]. Powdered ZnO–Au nanocomposites were synthesized via hydrothermal reactions, a simple, facile, and controlled method. ZnO nanorods were first grown and the rod surface was then decorated with Au NPs, resulting in ZnO nanorods with an average diameter of 300 nm and spherical Au NPs with sizes between 5 and 10 nm [46]. ZnO nanorod arrays were obtained by hydrothermal synthesis and then Ag NPs were deposited on the tips of the nanorods by photodeposition or galvanic reduction [47–48]. Another facile method included layer-by-layer self-assembly deposition of chemically synthesized Au NPs, ZnO, and analyte molecules and resulted in dispersedly distributed ZnO particles onto a Au NP monolayer surface [49].

One-step synthesis methods have been proposed for the fabrication of ZnO–metal nanocomposites as well, such as a fast one-step microwave assisted hydrothermal route [50]. Hollow doughnut-like ZnO–Au nanostructures were obtained, as well as other structures, including ZnO nanorods–Au NPs, ZnO nanodisks–Au NPs, and ZnO nanospheres–Au NPs, using this method. Some unusual fabrication methods include a poriferous ZnO–Ag substrate obtained by a film scraping method on paper [51–52]. A film with fluffy sponge-like morphology was obtained, pointing out its high specific surface area and showing uniform distribution of all elements. A more exotic strategy for developing SERS substrates based on ZnO and Ag was investigated by Chin et al. by employing a long-range π-conjugated segment, namely polydopamine, as an adhesive layer that can provide effective charge redistribution between the metal and semiconductor [53]. Additionally, the polydopamine layer assured a large number of functional groups, which served as anchors for controlled distribution and growth of Ag NPs, durability of the substrate, and biocompatibility.

Fewer studies paid attention to the reversed structural configuration based on coating metal NPs with ZnO. Au–ZnO core–shell NPs were obtained by preparing first AuNPs by chemical reduction and then fabricating Au–ZnO NPs by a seed growth method [51,54]. A higher enhancement in the Raman signal of *p*-aminothiophenol molecules was obtained using the Au–ZnO NPs compared to using Au NPs alone, an effect which the authors assigned to the amplification of the chemical effect of the ZnO layer by the LSPR of the Au cores. In another study, Ag nanowires were prepared by a polyol process and core–shell Ag–ZnO heteronanowires were synthesized by a simple solution process adding Zn(NO_3_)_2_ and milli-Q water to the freshly prepared Ag nanowire solution [55]. A higher photocatalytic activity was shown for the Ag–ZnO core–shell particles compared to ZnO alone under solar light irradiation.

### SERS applications of ZnO-based nanostructures

SERS is a powerful technique with promising applications for the detection of molecules [56]. Raman scattering in molecules is strongly amplified when they are located in the vicinity of nanostructured substrates [57], allowing for the ultrasensitive detection of target analytes down to subfemtomolar levels of concentration [54,58]. Other advantages of SERS include chemical specificity, non-destructive, and label-free detection. ZnO has been recently used for the development of SERS substrates [15] considering its properties, such as high refractive index, which can confine the excitation light in order to enhance the SERS effect, wide range of tuneable morphologies that can be used in combination with noble metals, but also its biocompatibility, photocatalytic self-cleaning capability, and high chemical stability.

The SERS effect occurs through the electromagnetic enhancement from the excitation of plasmon resonances in metallic nanoparticles and the chemical enhancement from the electronic interaction between the analyte and the nanosurface [59]. The electromagnetic enhancement factor (EF) can reach up to eleven orders of magnitude in the “hot spots” of the nanosubstrate [60–61], while the chemical EF usually has a value between 10 and 10^3^. Since the SERS activity of ZnO is weak [15] it can be improved by doping with heavy elements or by combining ZnO nanostructures with noble metals, thus increasing the electromagnetic or the chemical enhancement factor. Combined nanomaterials can offer increased SERS amplification due to both metal-induced EM and chemical enhancement owing to the interaction between semiconductor and/or noble metals with the analytes [7,35]. The chemical enhancement is related to the photoinduced charge transfer effect that takes place under the light excitation when the highest occupied molecular orbital (HOMO) and lowest unoccupied molecular orbital level (LUMO) of the analyte molecules match the conduction band (CB) and valence band (VB) of the semiconductor. The EM effect can be amplified by shifting the LSPR peak to the near infrared (NIR) or visible spectral region by doping the semiconductor and, thus, increasing the density of the free carriers or by creating “hot spots” on the ZnO nanosubstrates. For example, it has been observed that randomly oriented ZnO nanowires showed higher SERS enhancement than aligned ones [31], meaning that randomly oriented nanostructures may generate more “hot spots”.

One of the most relevant descriptors of any SERS substrate is the signal enhancement factor (EF), which describes the enhancement of the Raman signal of target molecules when adsorbed on the SERS substrate relative to the conventional Raman signal of the same number of molecules. The EF is generally calculated according to Equation 1:


[1] $$\text{EF}=\frac{I_{\text{SERS}}}{N_{\text{SERS}}}\cdot \frac{N_{\text{RS}}}{I_{\text{RS}}}$$

where *I*_SERS_ and *I*_RS_ are the SERS and Raman intensities, respectively, and *N*_SERS_ and *N*_RS_ are the number of excited molecules in SERS and Raman, respectively. The EFs are notoriously difficult to assess correctly due to a number of experimental and instrumental variables, such as the sample identity, number of molecules present in the probed region, as well as instrumental parameters. Nevertheless, certain good laboratory practices, proposed by Bell et al., can notably increase the consistency of EF estimations [62].

#### Electromagnetic and chemical enhancement using ZnO nanosubstrates

Previous simulations have shown that the Ag NPs exhibit the greatest plasmonic activity in the excitation wavelength range of 400–520 nm and the greatest absorption and electric field energy enhancement at the size of 50–60 nm, while for AuNPs these ranges are 525–580 and 90–100 nm (and potentially bigger), respectively [63]. For pure spherical Zn NPs, on average 28 ± 5 nm in diameter, obtained by vacuum magnetron sputtering on molten quartz, the plasmonic resonance is located around 240 and 290 nm, while for ZnO NPs, the maximum is around 360 nm [64]. In addition, ZnO nanosubstrates serve as carriers of Ag or Au NPs (or nanofilms), enabling a large area coverage with tiny and well-distributed Ag NPs, thereby efficiently exploiting their EM enhancement. Subsequently, both EM and charge-transfer (CT) enhancement contribute to the final SERS amplification [48], which is the reason for the use of ZnO nanosubstrates. By combining Ag or Au with ZnO into core–shell NPs it is possible to tune the resonance excitation of the whole system to shorter wavelengths, due to the overlap of multiple plasmonic resonance peaks arising from different metals. Resonant excitation can be shifted to longer wavelengths by prolonging one geometrical axis (nanorods) [63,65]. The same also holds for pure ZnO NPs, which are more often used as the core [64].

The non-plasmonic character of ZnO NPs in the visible range is convenient for more detailed studies of charge transfer mechanisms. Mao et al. used ZnO nanosubstrates in combination with Au and Ag plasmonic nanomaterials, the 514, 785, and 1064 nm laser excitation lines and *p*-aminothiophenol (PATP) as a molecule to study the charge transfer in metal–semiconductor–molecule–metal model systems [66]. They concluded that ZnO, with its work function (*W*_ZnO_) of 5.2 eV is in ohmic contact with Ag (*W*_Ag_ = 4.36 eV) and Au (*W*_Au_ = 5.1 eV), whereby charge transfer occurs in the direction ZnO–Ag or Au. Furthermore, the PATP spacer between ZnO and Ag acts to inhibit the charge transfer.

The study conducted by Zhou et al. [67] revealed the Raman signal of dopamine using Au–ZnO heterogeneous nanorods (NRs) and Au seeds alone. The results showed a stronger SERS signal in the case of Au–ZnO NRs compared to Au nanoscale seeds. The SERS signal enhancement is due to the increased charge transfer effect of ZnO, which is greatly improved by the localized surface plasmon resonance of Au seeds. For the detection of dopamine, an enhancement factor of Au–ZnO NRs greater than 1.2 × 10^4^ was obtained. The 3D SERS substrates based on Ag/ZnO/Au structures showed a higher SERS enhancement factor of 1 × 10^10^ and a limit of detection (LOD) down to 10^−16^ for rhodamine 6G (R6G) [68]. In this case, the charge transfer occurred from Ag and Au NPs to ZnO NRs. The 3D hybrid structures improve the surface-to-volume ratio, yielding a higher SERS activity. Moreover, the formation of “hotspots” and the Shottky barrier at the interface between ZnO NRs and the decorated Au NPs increases the SERS activity. Furthermore, limits of detection of 1.49 × 10^−13^ and 9.99 × 10^−12^ were reported by Kumar et al. for 4-nitrophenol (4-NP) and R6G, respectively, adsorbed onto a ZnO multipod suspension containing Ag nanospheres [69].

Increasing the sputtering time leads to an increase of the Au NPs size and a decrease of the interparticle gap, which is known to enhance the SERS signal. An optimum value for interparticle gap and size of Au NPs is needed to enhance the SERS signal. In [68] and [34], the fabrication of highly ordered flower-like ZnO nanorods decorated with Ag NPs is described. Sub-10 nm nanogaps between adjacent Ag NPs were the main electromagnetic “hot spots” that generated enhanced Raman signal. An EF of 10^6^–10^7^ was obtained for rhodamine 6G peaks [34]. Shape-controlled nanostructured Au–ZnO substrates obtained by photochemical reduction using various additives presented variable SERS intensities for rhodamine 6G detection. The SERS enhancement increased successively on Au spheres, Au NPs, dense Au NPs, conical Au, sea-urchin-like Au, and dendritic Au chains [32]. The highest SERS enhancement depended on both relative high coverage and plentiful gaps. Therefore, for Au NPs, despite the high Au coverage, lack of sufficient gaps resulted in low SERS enhancement, while dendritic Au showed the highest enhancement since it fulfilled both conditions.

It is clear that the quality of the ZnO-based SERS substrates depends on the coverage with metal nanoparticles. Ordered nanowires, one of the most commonly employed ZnO morphology for SERS measurements can be fabricated using various methods, including sol–gel synthesis [38], thermal deposition [31–32], chemical vapour deposition [42], or electrodeposition [35], which resulted in 1.2 μm high, vertically aligned nanorods of 60 nm diameter. It was observed that thermal annealing leads to ZnO NRs grown in the (002) direction and enhanced Raman signal. However, these types of NRs are not preferred for the growth of individual Au NPs used to further enhance the Raman signal, because the ZnO NRs tend to aggregate, which results in an inhomogeneous distribution of Au NPs on their surface. In contrast, ZnO NRs with horizontal (100) and (101) orientations were observed to promote the growth of Au NPs and their homogenous distribution, resulting in the formation of “hot spots” necessary for enhancing the Raman signal [38]. Also, ZnO nanoarrays obtained using atomic layer deposition followed by a modified low-temperature solution approach consisted of long ZnO whiskers and numerous stacked ZnO nanoflakes, with plenty of corners and edges where Ag NPs could be deposited. Further hydrogenation was introduced in order to increase the surface area of ZnO nanostructures and led to a higher adsorption of analyte molecules, increasing the EF from 10^6^ (before) to 10^8^ (after hydrogenation) [43]. The charge transfer effect was probably increased as well since the hydrogenation introduced lattice defects that could alter the energy band structure of ZnO, promoting charge separation at the ZnO–analyte or ZnO–Ag NP interface.

**Figure 3:**
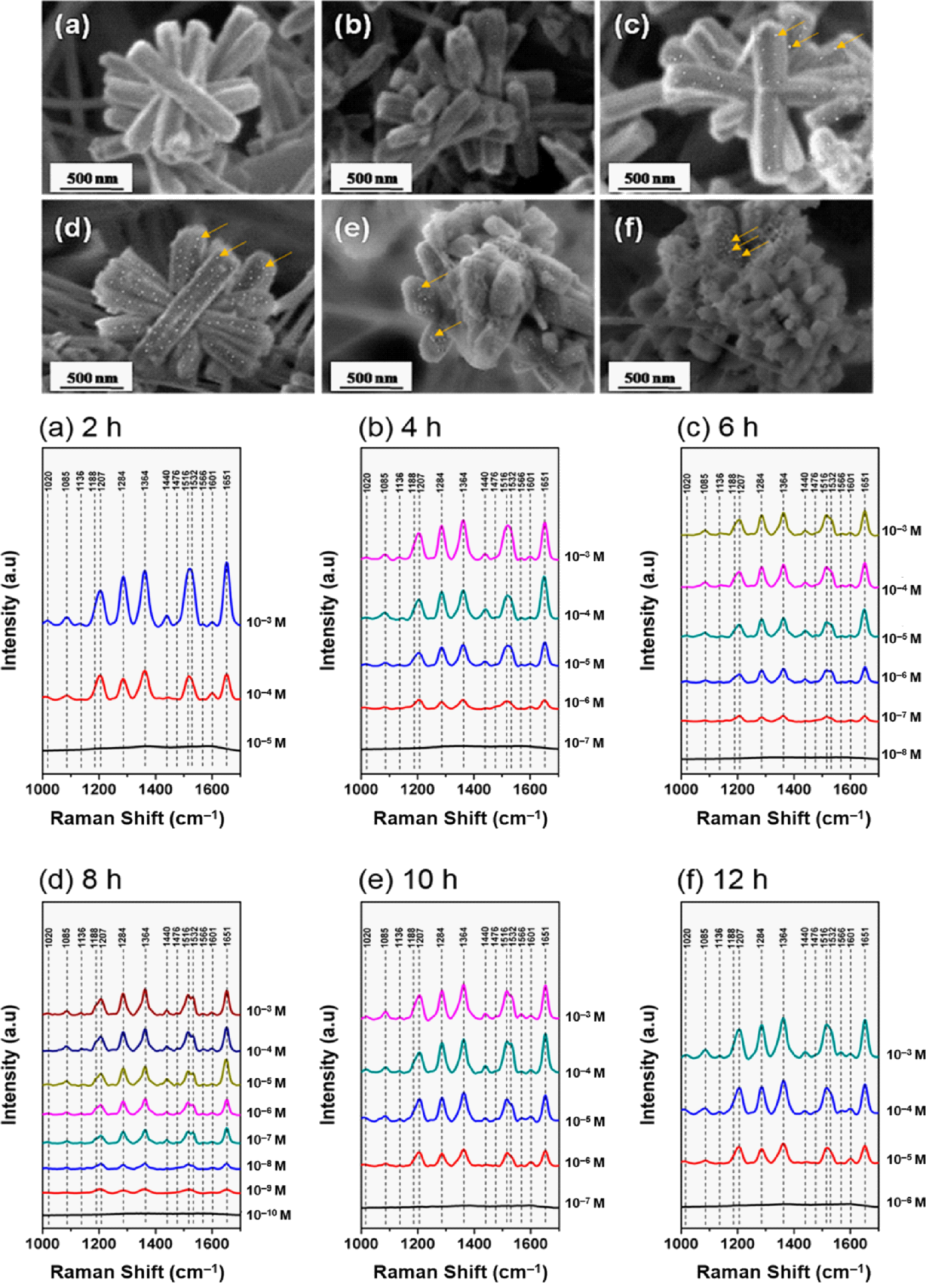
SEM images of silver nanoparticle–ZnO–polydopamine substrates and the SERS spectra of different rhodamine B concentrations acquired from each substrate. The deposition time of silver nanoparticles was varied for each substrate: (a) 2 h, (b) 4 h, (c) 6 h, (d) 8 h, (e) 10 h, and (f) 12 h. Figure 3 was adapted from [53] (© 2021 Chin, H.-K. et al., published by MDPI, distributed under the terms of the Creative Commons Attribution 4.0 International License, https://creativecommons.org/licenses/by/4.0).

Chin et al. [53] fabricated polydopamine-ZnO–based substrates using increasing Ag NP deposition times and observed a gradually decrease of the detected concentration of rhodamine 6G with increasing deposition time. However, for longer deposition times, which resulted in a high metal coverage of the ZnO nanorods, the limit of detection decreased (Figure 3). The large SERS enhancement factor of 10^10^ obtained for 8 h Ag NP deposition time was a result of the electromagnetic enhancement of the metal, the chemical enhancement and high refractive index of ZnO, which promoted strong light confinement, and the increased number of adsorption sites due to the branched ZnO nanostructures. Despite the fact that ZnO is a non-plasmonic semiconductor material, it can elicit a degree of SERS effect via chemical enhancement of atomic vibrational modes of the analyte. Kim et al. [70] reported that, from a theoretical point of view, there is no preferred charge transfer route between the non-plasmonic substrate and the analyte. However, the experimental observations on three different semiconductor–analyte systems showed that the charge transfer from the substrate valence band to the LUMO of the analyte is in fact the dominant process. For example, spherical ZnO NPs of 45–50 nm diameter showed SERS activity by themselves, detecting nanomolar concentrations of methyl orange and methylene blue [8].

**Figure 4:**
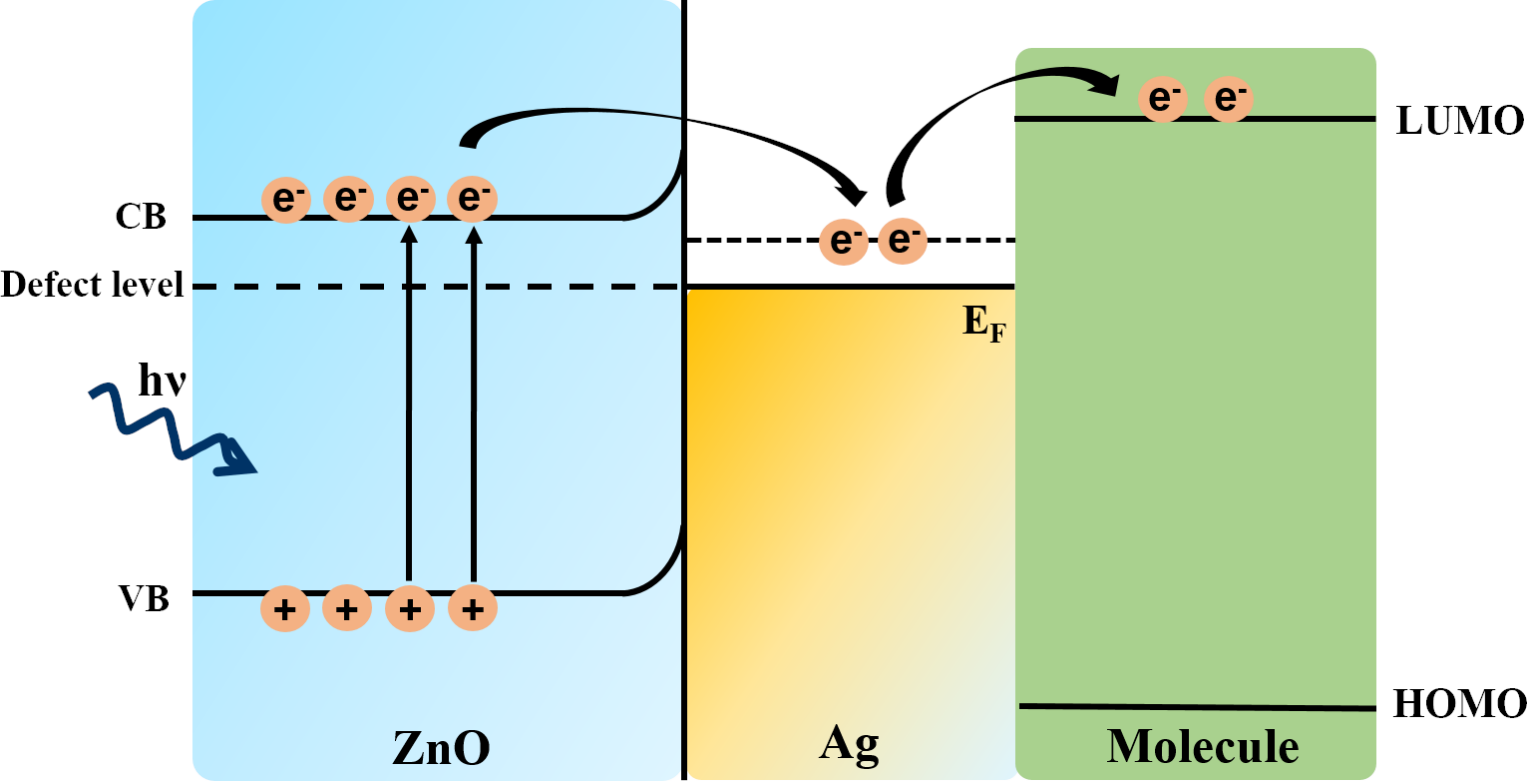
Schematic illustration of the charge transfer mechanism between ZnO, Ag, and the analyte molecule. VB: valence band, CB: conduction band, *E*_F_: Fermi level, HOMO: highest occupied molecular orbital, and LUMO: lowest unoccupied molecular orbital.

SERS effects can be obtained due to charge transfer between semiconductor (ZnO) and the metal. [71] showed that ZnO NRs with Ag-based substrates presented an enhancement of the Raman signal by acquiring a charge equilibration between the Fermi level of the metal and the CB of the ZnO. Excitation with visible light led to the following mechanism (Figure 4): The electrons from the VB of ZnO are excited to the CB, then the electrons are transferred into the Fermi level of Ag elevating it, and from here the electrons are transferred to the LUMO of the analyte molecules. Besides the existence of EM enhancement and CT enhancement due to Ag, the SPR effect of Ag leads to a decrease of the bandgap of ZnO. All of these are contributing to the SERS enhancement.

A SERS substrate enhancement factor of 5.4 × 10^7^ and an analytical enhancement factor of 1.3 × 10^10^ were obtained with Ag–ZnO heterostructures on glass substrates for the detection of methylene orange molecules. These SERS effect can be attributed to the EM enhancement due to clustering of Ag NPs and the CT mechanism [72]. [73] studied the SERS enhancement by using ZnO NRs decorated with Au NPs. Deposition of gold NPs onto ZnO nanorods resulted in decreasing the green emission band and enhancing the exciton emission band. According to the authors, one of the reasons for this phenomenon was the coupling between surface plasmon absorption of Au NPs and photoluminescence of ZnO nanorods. However, the ZnO–Au nanostructures showed a high SERS activity, demonstrated for methylene blue (MB) with a limit of detection of approximately 10^−9^ M. Moreover, the study in [74] found a promising enhancement factor for a hollow ZnO–Ag nanosphere SERS substrate of 3.17 × 10^8^ and a limit of detection of 0.3 × 10^−8^ M for nitrite species.

#### Recyclable ZnO nanosubstrates

**Figure 5:**
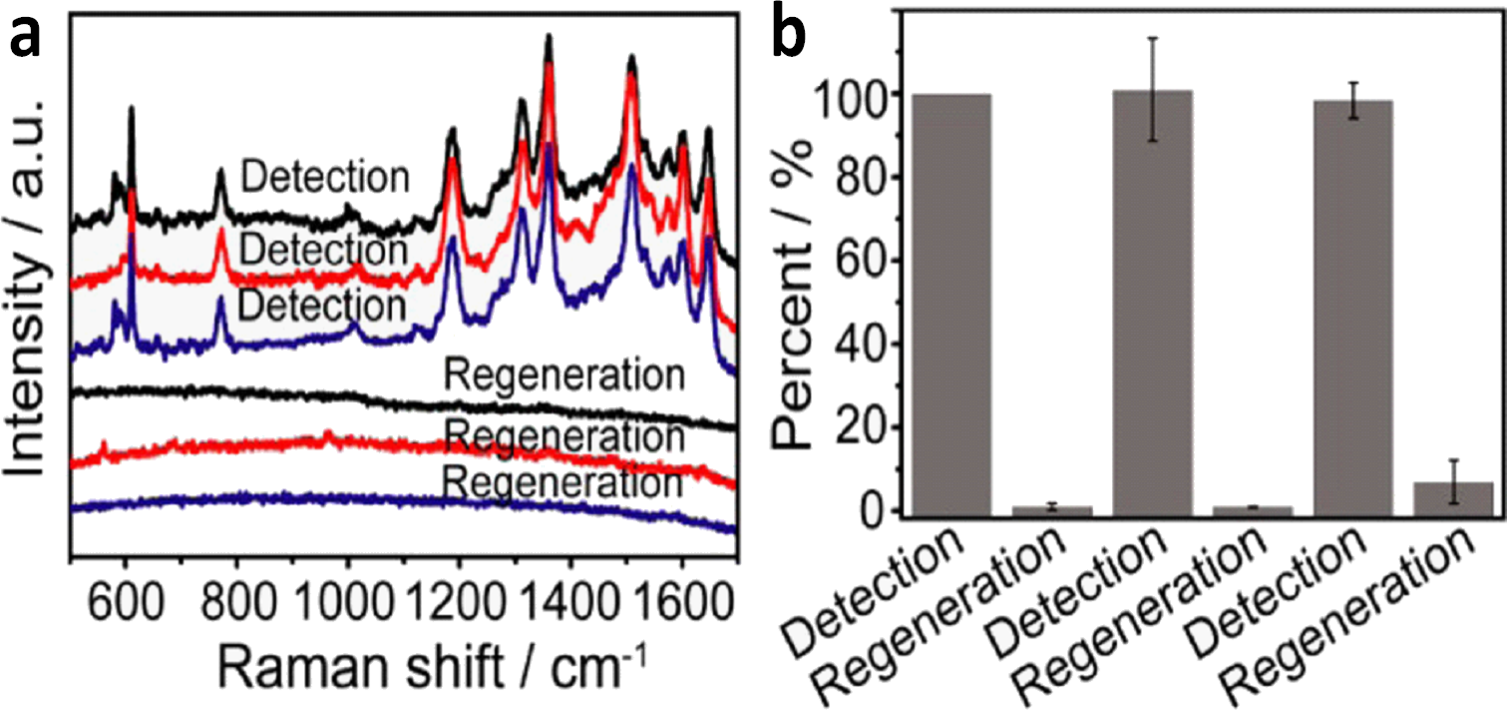
Recyclability demonstrated for an Au–ZnO substrate. SERS detection (a) of rhodamine 6G and regeneration of the substrate. Each coloured line represents a cycle of detection and photocatalytic cleaning of the substrate. (b) Percentage of degradation and recovery calculated for the 1360 cm^−1^ Raman band of rhodamine 6G. Adapted with permission from [32] Copyright (2016) American Chemical Society. This content is not subject to CC BY 4.0.

Another motivating advantage of the ZnO-based plasmonic substrates refers to their recyclability. A renewable substrate is obtained when target molecules adsorbed on it can be fully removed and the substrate reused. Since metal-based SERS substrates are disposable after being used, the photocatalytic property of ZnO would ensure reduced consumption of resources and costs when used as a SERS substrate. The self-cleaning ability of ZnO alone or ZnO–noble metal-based nanostructured substrates has been shown under UV or visible irradiation via the photocatalytic degradation of the organic molecules attached to the substrate [8,34,53]. For instance, [75] presented Au-coated roughened ZnO nanostructures, which enabled photocatalytic degradation of adsorbed analytes and the reuse of the substrate. Also, [73] demonstrated the self-cleaning ability of ZnO–Au nanostructures under UV irradiation. The rhodamine 6G adsorbed on Ag–ZnO–Au film showed a fast photocatalytic degradation due to higher surface area and the presence of a metal–semiconductor junction by this 3D hybrid structure [68]. Additionally, it was observed that metal-based substrates did not exhibit any catalytic degradation, while those that incorporated ZnO led to a significant decrease in the SERS intensity under the same irradiation conditions [53]. A typical experiment to test the reusability of an Au–ZnO substrate consisted of immersing the substrate in a dye solution for 12 h and characterizing it by SERS. Afterwards, the substrate is immersed in ultrapure water and exposed to UV irradiation for 1 h to remove the analyte. Figure 5 shows the disappearance of the SERS signals of rhodamine 6G after UV irradiation and the possibility to reuse the substrate for several cycles [32].

It appears that the photocatalytic degradation depends on the geometrical configuration of the ZnO–metal nanocomposites, as well as the energy of the incident light [36]. Moreover, the substrates could be used after several recycling cycles and presented similar EFs as the fresh ones. Other methods for recycling the ZnO-based nanosubstrates include thermal treatment and magnetic separation. These benefits indicate that the development of composite semiconductor–noble metal-based substrates is desirable since they could be implemented as reusable, low-cost SERS substrates for ultrasensitive detection of analytes.

#### ZnO lattice defects and doping

ZnO nanostructures usually present two emission bands, namely a narrow UV band assigned to exciton recombination [76] with a maximum around 370 nm (3.35 eV), called band-edge luminescence and a broad emission band situated in the green spectral region at about 550 nm (2.25 eV) in undoped ZnO [77]. The nature of the long-wavelength band in the visible region has been controversial for a long time. It was assumed that zinc vacancies, oxygen vacancies, and other defects are candidates for recombination centres involved in the visible luminescence [78]. Studies concerning the green luminescence of ZnO in the past few years assumed that the oxygen vacancies are the most likely candidates for recombination centres involved in the visible luminescence of ZnO [77,79]. Thus, the electrical and luminescence characteristics of zinc oxide could be changed by the number of oxygen vacancies or other point defects (vacancies, atom substitutions, and interstitial atoms). Moreover, introducing point defects in the ZnO lattice enables an efficient charge transfer, which enhances the SERS signal. [80] demonstrated that heat treatment in an oxygen atmosphere is an effective method for the introduction of interstitial oxygen point defects in the ZnO lattice, which leads to SERS enhancement. Introducing defects reduces the optical gap, which allows for a more effective charge transfer between ZnO–Ag nanowires and the molecules attached. Also, the interstitial oxygen defects change the wettability of ZnO–Ag nanowires, which reduces the spreading of Ag NPs or probe molecules on the surface, which also benefits the SERS enhancement. Doping zinc oxide–Ag nanoparticles with magnesium also introduces defect sites (surface defects and oxygen vacancies), which form new energy levels below the conduction band of zinc oxide, facilitating the charge transfer mechanism. In this case, besides the charge transfer contribution to SERS signal enhancement, the electromagnetic contribution also has an impact on the SERS signal [81].

Xue et al. [82] and Libin Yang et al. [83] argued that ZnO lattice defects created by doping the nanoparticle lattice with other cations increases the SERS enhancement by promoting electron transfer and creating sites for the binding of electrons from surface-state energy levels, followed by further transfer of the electrons to the LUMO of the adsorbed molecules. However, it should be noted that too high doping/defect concentrations are not desirable, as they can cause electron–hole recombination competing with the charge transfer from nanoparticles to molecules. For instance, [82] observed that 1% doping of ZnO NPs with Co provides the strongest SERS enhancement of 4-MBA, 4-MPY, and PATP signals, while either greater or lesser extents of doping result in weaker enhancement; [83] came to a similar conclusion with 3% doping of TiO_2_ NPs with Zn, using 4-MBA as analyte.

### Fluorescence applications of ZnO-based nanostructures

#### Photoluminescence enhancement of ZnO nanostructures

ZnO nanomaterials present a wide bandgap of (3.3–3.7 eV) and a high excitation binding energy of 60 meV, which gives them promising optoelectronic device applications [84–85]. Being a wide-bandgap n-type semiconductor, ZnO can give a strong UV emission even at room temperature. Thus, it presents high photocatalytic activity and the potential to use it for UV light emitters. Apart from UV emission, the photoluminescence (PL) spectrum of ZnO also consists of a broad band emission in the visible region, which is generally assigned to defect levels [86–87]. The combination of ZnO nanostructures with metal nanoparticles can result in new optical features and the development of a new class of multifunctional optical components, due to the energy and electron transfer from plasmon-resonant metal surfaces to the adjacent semiconductor. Such hybrid materials have been proposed for medical and pharmaceutical applications, catalysis, and electronics [88]. The photoluminescence emission of ZnO nanoparticles has been exploited in applications for optoelectronic devices, namely light-emitting diodes for both UV and visible light [89]. Also, the combination with metallic nanoparticles has been proposed for enhanced UV light-emitting diodes [90]. Another significant application of ZnO-based substrates is to boost the performance of dye-sensitized solar cells (DSSCs). Au–ZnO plasmonic nanoparticles were studied for DSSCs application and their enhancement capabilities were assigned to the SPR effect [91].

**Figure 6:**
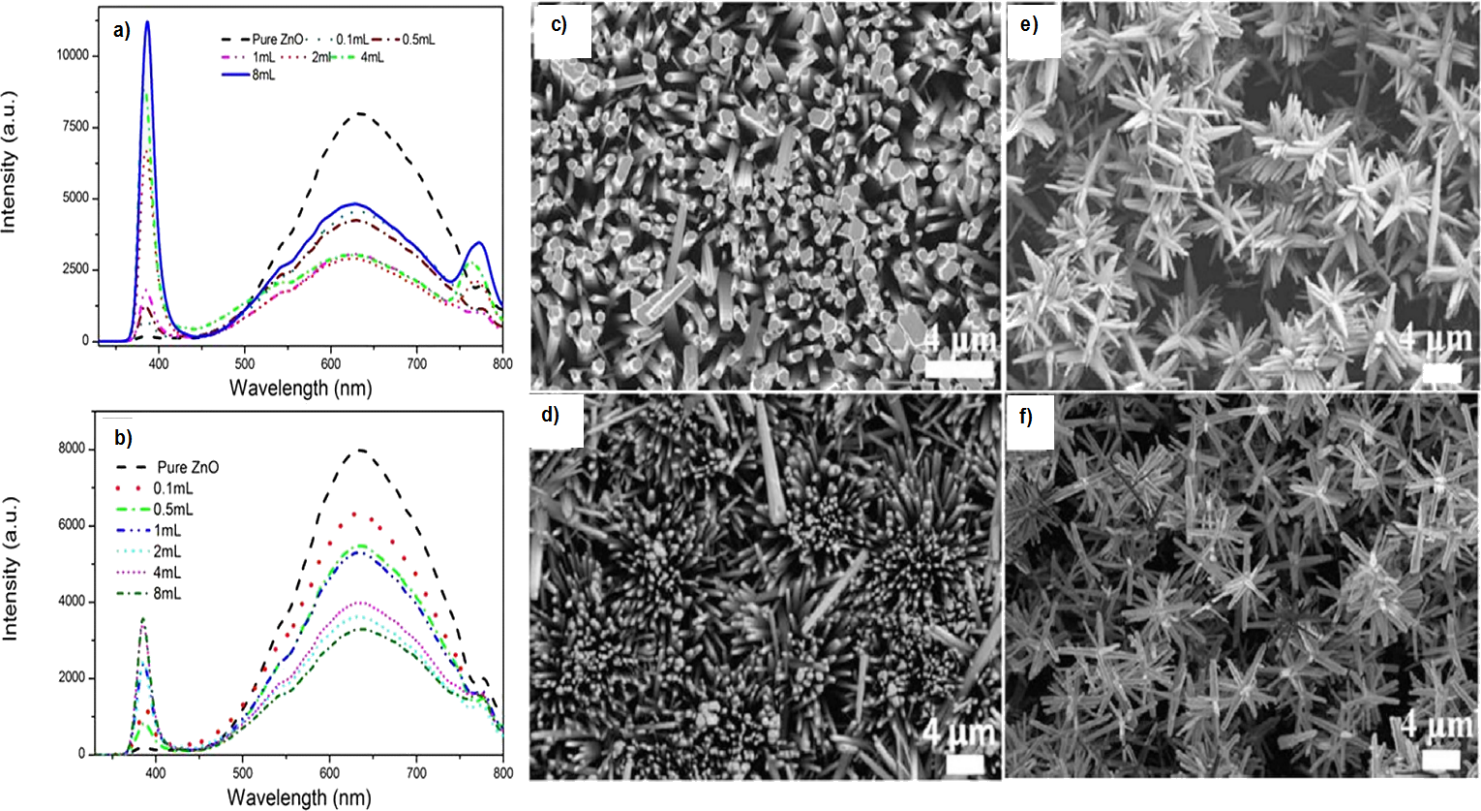
Photoluminescence spectra of ZnO–Ag (a) and ZnO–Au (b) shell–core nanoparticles obtained with 325 nm excitation. SEM of ZnO nanorods (c) and core–shell ZnO–metal nanostructures obtained with different amounts of metal nanoparticles: 2 mL Ag NPs (d), 8 mL Ag NPs (e), and 8 mL Au NPs (f). Figure 6 was adapted from [88] (© 2015 Guidelli, E. J. et al., published by SpringerNature, distributed under the terms of the Creative Commons Attribution 4.0 International License, https://creativecommons.org/licenses/by/4.0).

Hence, the ZnO PL is of great interest and its enhancement is also important in order to develop efficient optoelectronic devices. Numerous studies have demonstrated the enhancement of both near band edge (NBE) and defect emissions using ZnO decorated with metallic NPs [92–93]. The study in [92] showed a PL enhancement of the Au–ZnO heterojunction system by an order of magnitude when ZnO thin films were incorporated with gold nanoislands. The enhancement is considered to be attributed to a hot carrier transfer from Au to ZnO. Contradictory, Brewster et al. observed a 32% decrease in UV emission intensity after Au NP decoration of ZnO [93]. This quenching effect was assigned to electron transfer from the ZnO conduction band to the Au NPs. Photoluminescence spectra of ZnO films were studied after doping them with 29% Au annealed at 300 °C in air. After Au incorporation, a strong increase of both UV and visible ZnO emission bands was obtained [94]. Another study [95] obtained 1.5-fold to 2.8-fold enhancements for Au-decorated ZnO microrods, depending on the incident power of the green light excitation. The NBE emission was strongly enhanced, while the defect band emission decreased simultaneously. This behaviour after ZnO decoration with Au NPs is assigned to localized surface plasmon coupling between Au and ZnO. Some examples of NBE emission enhancement include ZnO nanorods surface decoration with Ag NPs [96] or Au NP-decorated ZnO nanoflowers [97]. A 60-fold and, respectively, a 17-fold enhancement of the NBE emission of ZnO was achieved with shell–core ZnO–Ag and ZnO–Au NPs, respectively [88]. While the NBE emission was strongly increased, the defect band intensity was reduced (Figure 6). The observed enhancements were assigned to modifications in the creation of excitons and their recombination lifetimes, more specifically an increase in the non-radiative transitions and a decrease of the radiative emission of ZnO. Time-resolved fluorescence measurements showed that increasing the Ag NP concentration in hybrid metal–ZnO nanocomposites resulted in an increase of non-radiative transitions and a decrease of the radiative emission of ZnO, indicating the energy transfer from ZnO excitons to the Ag NPs plasmons [88].

Additionally, the features of ZnO nanostructures photoluminescence spectra were shown to depend on the thicknesses of the Au layer. Viter et al. obtained interesting results studying the variation of photoluminescence spectra with the thickness of the Au layer in Au–ZnO thin films. Using a thin layer of Au (5 nm), the strong UV peak emission was enhanced and the visible emission was quenched. In contrast, when using a thick layer of Au (30 nm), the UV emission was drastically reduced, while the visible emission was enhanced [98].

**Figure 7:**
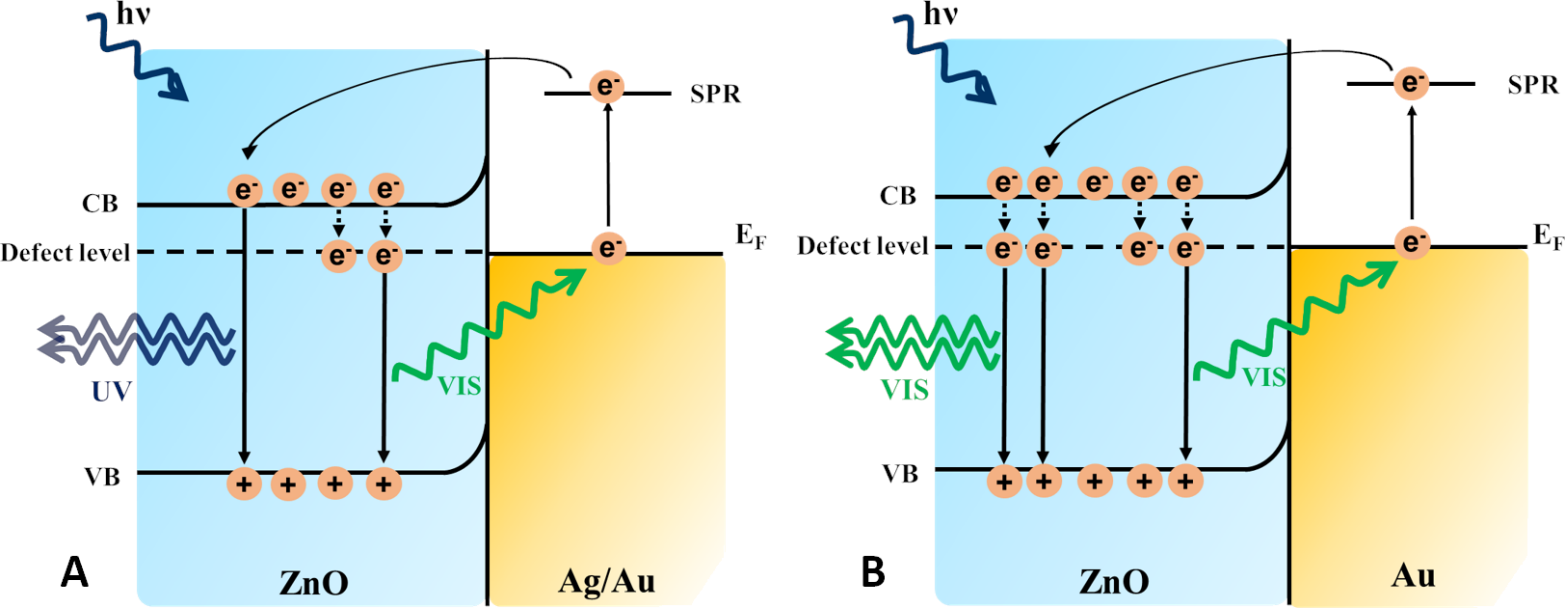
Schematic illustration of the mechanism for: a) UV emission enhancement of Ag NPs or Au NPs decorated ZnO and b) visible emission enhancement of Au NPs decorated ZnO. VB-valence band; CB-conduction band; *E*_F_-Fermi level; SPR-surface plasmon resonance level.

The PL enhancement has been assigned to the localized surface plasmon resonance of metallic nanostructures [99–100], as well as to charge transfer-induced electron–hole recombination. In the case of metal NP-decorated ZnO NRs [96,101], the UV emission enhancement mechanism is attributed to the recombination between holes in the VB and the electrons in the CB, while the visible-range emission is induced by recombination between holes and the electrons in a deep defect level. This defect emission energy matches with the surface plasmon absorbance energy of Au NPs; therefore, it can absorb the visible-emission energy of ZnO NRs. The exact mechanism is described in Figure 7A based on the description in [101]. First, the ZnO is excited and because most of the electrons are trapped in the defect level of ZnO NRs, they will recombine with the holes in the VB resulting in a visible-range emission. In the case of contact between ZnO NRs and Au NPs, the Au NPs can absorb the visible-emission energy, transferring the electrons to a higher energy state. Second, because the work function of ZnO is 5.2 eV and the work function of Au is 5.1 eV, ZnO can receive energy from Au until these two systems achieve a dynamic equilibrium. Thus, the electrons are transferred into the CB of ZnO NRs and the recombination between electrons and holes in the VB of ZnO NRs takes place. The result is a great enhancement of the UV band emission and a suppression of the defect emission of ZnO NRs. The mechanism for UV emission enhancement of Ag NPs decorated ZnO NRs is similar to the one described above for Au NPs decorated ZnO NRs (Figure 7A).

Besides the UV emission enhancement of ZnO NPs by metal NPs, the presence of metal could also enhance the green emission. The green emission enhancement of Au-decorated ZnO NRs was observed for a higher concentration of Au, which increased single ionized oxygen vacancies (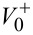). The green emission is correlated with 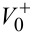 vacancies, which capture the electrons from the conduction band turning into a metastable state. The recombination of these captured electrons and the holes in the VB lead to the green emission [102]. Figure 7B shows the mechanism behind the green emission enhancement of ZnO, which is associated with a strong local field enhancement generated by the LSPR of Au NPs [103]. After the excitation of ZnO, visible emission occurs and generates surface plasmon oscillations in the Au NPs attached to ZnO NRs. The electrons from the excited states can be transferred to the conduction band of ZnO. The 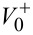 defects capture electrons from the conduction band and their recombination with the holes from VB can result in the green emission that dominates the spectrum at higher Au concentration attached to ZnO NRs. A 7-fold enhancement of ZnO defect emission was demonstrated for Au–ZnO nanocomposites obtained through a simple photoreduction method and has been assigned to the surface plasmon oscillations of Au and the electron capture by the single ionized oxygen vacancies in ZnO [103].

#### Surface-enhanced fluorescence using ZnO nanostructures

Fluorescence detection is a powerful and effective tool for monitoring compounds with native fluorescence or labelled with an appropriate tag. For fluorescence-based detection, achieving high sensitivity via improving the signal-to-noise ratio is mandatory [104]. In order to increase the quantum yield and, consequently, to increase the sensitivity of fluorophores, the use of metallic nanostructures was proposed due to their localized surface plasmon resonance [105]. The improvement in the fluorescence detection efficiency can be achieved using fluorophores in the proximity of nanosubstrates, a technique named surface-enhanced fluorescence (SEF). This method is based on designing nanosurfaces and then using them near the emitter in order to control its local electromagnetic environment [106]. The molecules that are in the vicinity of nanosubstrates or adsorbed to their surface suffer a modification of their fluorescence intensities and lifetimes, leading to either quenching or enhancement of their fluorescence signal [107]. The SEF technique is also called metal-enhanced fluorescence (MEF) when the fluorophore interacts with metal nanostructures. MEF originates from the coupling between the dipole moment of the fluorophore and the surface plasmon field of the metallic nanostructure. This interaction leads to the enhancement of fluorophore emission, being an adequate method to improve the sensitivity of fluorescence detection of molecules. Based on the above, MEF is also referred to as plasmon-enhanced fluorescence (PEF). The excitation of localized surface plasmon resonances in the metal nanostructures can lead to an enhancement of the local field and emission intensity. The coupling between the confined field of surface plasmons of the metal nanostructure with the emission spectra of the fluorophores is considered to enhance the fluorescence signal [105,108].

Enhancement and quenching of fluorescence depend strongly on the distance between the fluorophores and the metal surface [109]. In the case of metal-based substrates, fluorophores should be situated between 5–90 nm [110–111] away from the metal surface in order to facilitate MEF. Instead, nanostructured ZnO substrates do not show such a distance-sensitive effect, therefore, they do not induce fluorescence quenching from this point of view [112]. Even if the fluorophores are placed directly onto the ZnO nanorods surface, no quenching effect has been detected [113]. ZnO platforms are inexpensive to produce, non-toxic and biodegradable, showing potential for improving the detection of various molecules as fluorophores, dyes, or cancer biomarkers. Thus, the use of ZnO NPs as substrates for fluorescence enhancement is an alternative to overcome the shortages of metal-based substrates, such as fabrication cost and susceptibility to fluorescence quenching.

Another significant advantage of using ZnO-based substrates is their high capacity for multiplex detection, attributed to their potential to significantly enhance the fluorescence over a broad range of wavelengths. This multiplexing property was investigated for ZnO nanoflower (NF) and nanorod arrays by [114]. Different Alexa Fluor (AF) dyes were used and the results showed a significant enhancement of their fluorescence over the entire visual spectral range (400–840 nm). Specifically, using ZnO NFs a great fluorescence enhancement factor of up to 45 for AF 647 over the spectral range 660 to 750 nm was obtained. It was demonstrated that fluorescence enhancement is geometry-dependent. For a large diameter, fluorescence is improved and it was shown for both ZnO NR forests and NFs. A more significant enhancement was obtained for NFs, which also presented tuneable fluorescence enhancement [114].

**Figure 8:**
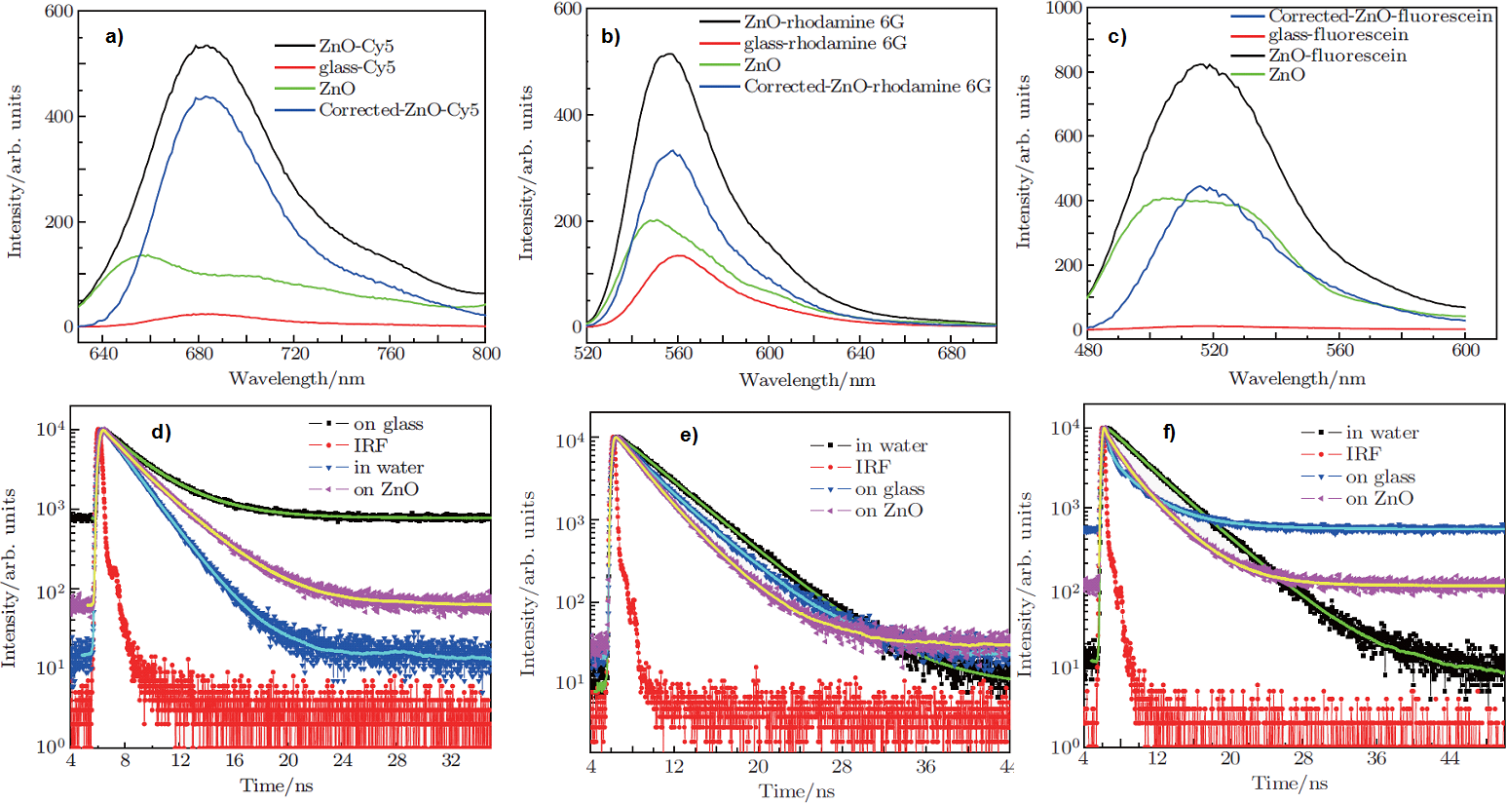
Fluorescence emission and kinetic traces characteristic to Cy5 (a and d), rhodamine 6G (b and e), and fluorescein (c and f) water solutions and to the respective spin-coated dye solutions on glass substrates with nanostructured ZnO films. Figure 8 was adapted from [115] (Y.-S. Liu et al., “Nanoscaled ZnO films used as enhanced substrates for fluorescence detection of dyes”, Chin. Phys. B, vol. 21, article no. 037803, published on 7 October 2011; https://doi.org/10.1088/1674-1056/21/3/037803); © 2012 Chinese Physical Society and IOP Publishing Ltd. Reproduced with permission. All rights reserved. This content is not subject to CC BY 4.0.

A 10-fold fluorescence enhancement was detected for Cy5, rhodamine 6G, and fluorescein placed on a nanoscaled ZnO film [115] (Figure 8a–c). The fluorescence enhancement is only related to the larger surface area of the ZnO nanostructures. It was determined that using nanoscaled ZnO films did not cause any significant modification in the decay rates on the radiative pathways of fluorophores as compared to those on glass (Figure 8d–f), indicating that the mechanism of enhanced fluorescence based on ZnO nanoscaled films is different from the metal enhancement.

The use of ZnO nanosubstrates for enhanced biomedical detection has received great attention due to their optical properties and other great advantages. Early detection of disease markers and the measurement of specific protein concentrations is a key tool in clinical medicine because it can provide a better diagnostic tool. Fluorescence is also broadly used in biotechnology as a sensitive detection method for different molecules. For all of these applications, high fluorescence quantum yield, photostability, and brightness of the used fluorescent molecules are essential. The values of quantum yield and lifetime could be influenced by non-radiative decay rates, which result from changes in the environment of the fluorophores, fluorescence quenching, or fluorescence resonance energy transfer (FRET) [112,116]. Engineered ZnO nanosubstrates were successfully used for the detection of relevant biological and biomedical proteins, such as bovine serum albumin and streptavidin, as well as to study the protein–protein interactions by enhanced fluorescence detection [117]. The ZnO nanoplatforms showed several key advantages, such as enhanced fluorescence emission of fluorophores emitting at different wavelengths, chemical inertness to the environmen, and simple assembly and fabrication. Moreover, the ZnO nanoplatforms can be covalently modified to host specific protein molecules of interest only and to present increased detection specificity of protein reactions. The study demonstrated that ZnO substrates can be employed as efficient nanoplatforms for rapid identification and screening of interacting protein pairs. A significant fluorescence amplification was reported for ZnO NRs substrates for the detection of two important cancer biomarkers (carcinoembryonic antigen and α-fetoprotein), achieving a detection limit of 1 pg/mL in human serum [51]. The authors, however, could not explain the mechanism responsible for enhanced fluorescence signals in the presence of ZnO nanosubstrates. There are works that studied the mechanism of fluorescence enhancement of molecules in contact with nanostructured ZnO substrates. For example, [117] concluded that the surface area of ZnO or protein coverage does not influence the fluorescence enhancement, but is related to the intrinsic properties of ZnO. They also noted that the enhanced fluorescence could be explained by variations in photonic mode density and/or reductions in the self-quenching of fluorophores, which was attributed to the presence of traps in the energy levels of the fluorophores [104,118]. Moreover, [113] discussed two pathways responsible for enhanced fluorescence signals of ZnO NRs. The first mechanism believed to be responsible for fluorescence enhancement is based on reducing the resonance energy transfer between fluorophores, which will prevent self-quenching. ZnO NRs have the ability to guide light [119] in and out of fluorophores and along nanorods and the ability to enhance the intensity of the evanescent field. These properties explain the second mechanism of fluorescence enhancement of ZnO NRs. Regardless of the origin of fluorescence enhancement, ZnO nanosubstrates proved to be economical and sensitive arrays that could be mass-produced for broad applications in clinic diagnosis, therapeutic monitoring, and drug discovery [51]. Several examples of fluorescence enhancement using ZnO nanostructures are given in Table 1.

**Table 1:** Examples of fluorescence enhancement using ZnO nanostructures.

Configuration of ZnO nanostructures	Diameter/size	Molecules	Fluorescence enhancement	Ref.

nanorods	200 to 500 nm	carcinoembryonic antigen and α-fetoprotein	20-fold	[51]
films	—	Cy5, rhodamine 6G and fluorescein	10-fold	[115]
nanorods	53 to 93 nm	Alexa Fluor 700 (AF700)	EF from 3 to 7 (is a function of diameter)	[120]
nanoflower	4 to 72 nm	Alexa Fluor 700 (AF700)	EF from 19 to 29 (is a function of diameter)	
nanoflower	718.5 ± 46.9 nm	AF 647	EF up to 45	[114]
nanoplatforms	square length of 10 μm	fluorescein-conjugated antibovine IgG (FITC-anti IgG)	—	[117]
nanorods	the average diameter and length are 180 ± 12 nm and 1.2 ± 0.3 μm, respectively	cytokines interleukin-18 (IL-18) and tumor necrosis factor-α (TNF- α)	—	[113]
nanotubes	average outside diameter 12 nm	fluorescein isothiocyanate (FITC)	90-fold	[121]

Fewer studies investigated the enhancement of fluorescence in ZnO–metal nanocomposites and further research is still needed to explore this direction. Core–shell metal–ZnO nanocomposites have shown new exciting properties and even metal-enhanced fluorescence for dyes mixed with the NPs. [20] demonstrated MEF for Ag–ZnO using rhodamine 6G (R6G), prepared through a simple chemical reduction method and deposition of the nanoscale ZnO layer on the surface of Ag NPs. The MEF performance was investigated for different growth temperatures of the nanoscale ZnO layer and a fluorescence increase followed by a decrease was observed with increasing the temperature. Also, the fluorescence emission intensity of R6G molecules varied with the surface area of Ag–ZnO nanocomposites. The influence of annealing temperature and annealing time for the MEF property of Ag–ZnO composite structure was also studied using fluorescein isothiocyanate (FITC) [122]. The fluorescence emission of FITC showed an increase at higher values of annealing temperature or annealing time. The improvement in fluorescence efficiency was attributed to the large distance between different FITC molecules on the surface of the Ag–ZnO composite, which reduced the self-quenching effect. Core–shell Ag–ZnO nanocomposites showed a R6G fluorescence enhancement of 53% compared to the signal from the same amount of R6G without NPs [123]. The fluorescence enhancement was attributed to the ZnO layer, which ensured a distance between the metal core and the fluorophore molecules. However, the enhancement was also possibly due to the ZnO layer since it could change the photonic mode density, as well as reduce the self-quenching of R6G.

**Figure 9:**
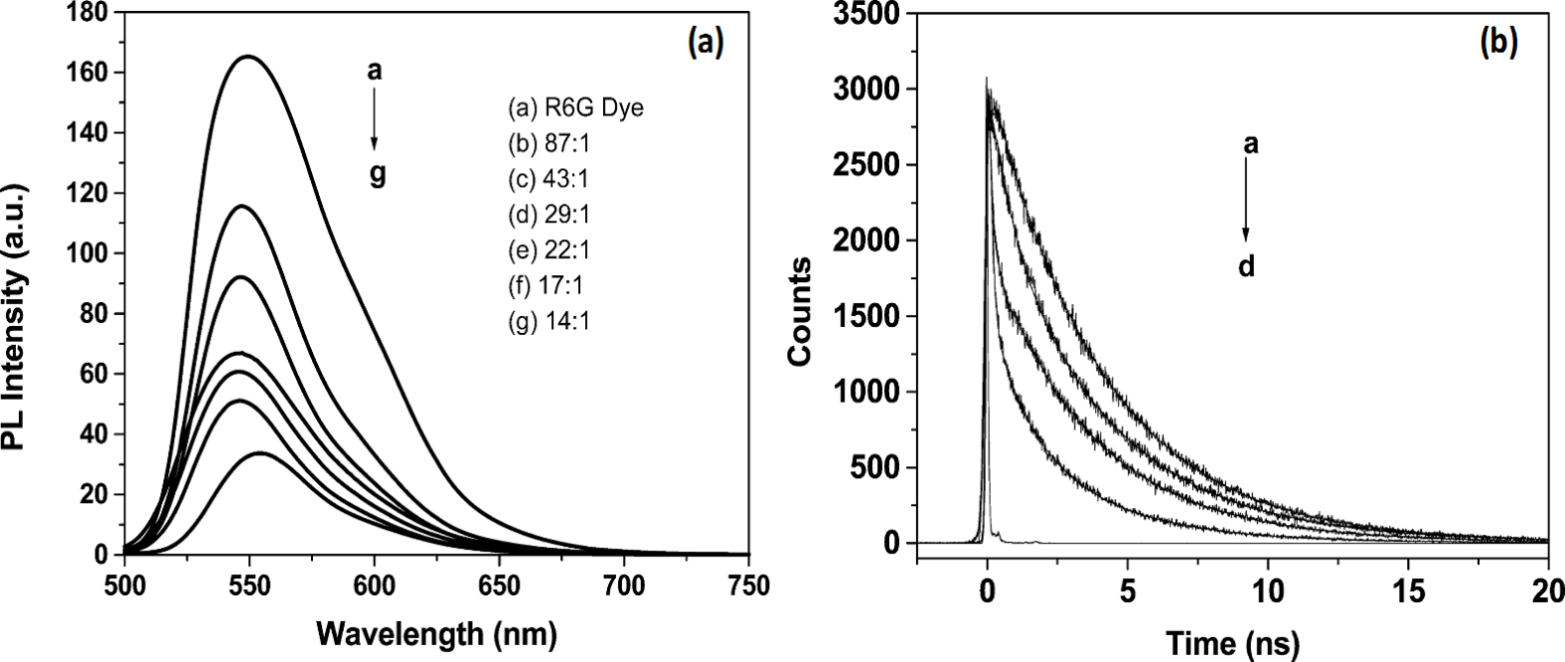
Fluorescence spectra (a) of rhodamine 6G in the presence of Au–ZnO nanostructures and kinetic traces (b) of rhodamine 6G dye solution, Au and 1 μM R6G, Au and ZnO nanoparticle mixture and 1 μM R6G, and Au–ZnO core–shell with 1 μM R6G. Adapted with permission from [125] Copyright (2008) American Chemical Society. This content is not subject to CC BY 4.0.

Considering the fluorescence enhancing properties of ZnO NPs, FRET studies have been carried out as well. FRET, which is a non-radiative process based on the energy transfer between an excited state donor and a ground state acceptor, can be manipulated using NPs, since it depends on a number of parameters including fluorescence lifetime and quantum yield of the donor molecule. The FRET effect has been investigated between two dyes, fluorescein, and rhodamine 6G, in the presence of ZnO nanostructures. An increase of FRET (69%) in the presence of ZnO flower-shaped NPs was observed for concentrations between 10^−6^ and 10^−5^ M of both the fluorophores and ZnO NPs [124]. The ZnO NPs were responsible for an enhanced fluorescence emission of fluorescein (donor in the investigated system), by allowing for an increased energy transfer efficiency. Moreover, the FRET efficiency increased with pH, suggesting the possible use of the system as a FRET-based pH sensor. The FRET effect was investigated for core–shell Au–ZnO nanoparticles as well in the presence of rhodamine 6G dye. The results showed an efficient energy transfer of 72.6% from the dye to the core–shell Au–ZnO nanoparticles, which was confirmed by the quenching process (Figure 9a) and the shortening of fluorescence lifetime of the dye (Figure 9b), indicating a high charge storage capacity. The photoluminescence quenching of the dye was attributed to the non-radiative decay pathways, which confirmed FRET between the dye and core–shell Au-covered ZnO nanoparticles [125].

## Conclusion

The promising characteristics of ZnO nanomaterials have recently propelled their investigation for a wide range of applications in optical sensors and optoelectronic devices. This review focused on their applications in surface-enhanced Raman spectroscopy and surface-enhanced fluorescence, two powerful analytical tools based on nanosensors for the sensitive detection of chemical and biological molecules. ZnO alone has presented appealing enhancing abilities in fluorescence-based detection applications. However, in the case of SERS, the performance is still moderate. An attractive solution for improving the sensing capabilities of ZnO nanostructures relies on the development of hybrid nanomaterials based on ZnO and metal nanoparticles. In this manner, the optical properties of ZnO can be improved and adjusted according to the targeted application due to the excitation of localized surface plasmon resonances. ZnO nanostructures can be designed into various controlled morphologies and sizes owing to the advancement in fabrication techniques. Moreover, facile and low-cost synthesis methods can be adapted for large-scale production. The fabrication techniques employed to develop ordered hybrid nanostructured substrates range from more expensive and laborious ones to more cost-efficient and simple ones. The main challenge of fabrication methods relies in the simultaneous development of ordered ZnO nanostructures with sufficient metal coverage and in the creation of “hot spots” at the surface of ZnO nanoparticles required for the amplification of the Raman signal. Therefore, the current demand of metal–semiconductor nanocomposites for nanoscale sensor devices requires the development of more facile and versatile synthesis routes that can yield the required fabrication and performance conditions.

The ZnO–metal nanomaterials have shown increased SERS activity compared to ZnO alone or metal-based nanosubstrates. The performance enhancement requires the existence of both electromagnetic and charge transfer enhancements, which can be accomplished by the controlled tuning of the surface plasmon band of the nanosubstrate to the visible region and ensuring efficient interaction of the semiconductor and/or noble metals with the analytes. All of these are essentially contributing to the SERS enhancement. Despite some of the current fabrication challenges and still limited sensing applications, the recently obtained enhancement factors indicate that ZnO-based nanosubstrates are able to achieve high selectivity and ultrahigh sensitivity. Moreover, several encouraging properties of ZnO, such as biocompatibility and self-cleaning, have yet to be further researched. ZnO-based substrates show a photocatalytic potential to degrade the organic molecules attached to their surface and ensure the reproduction of the Raman signal after being cleaned. By incorporating metal NPs, the photocatalytic performance can be enhanced, thus, ensuring economic and environmental advantages.

The strong luminescence of ZnO has guaranteed its exploitation for optoelectronic devices, namely light-emitting diodes. Recent research on the fluorescence enhancing capabilities of combined ZnO–metal nanostructures, however, has resulted in contradictory results. Therefore, further studies are needed in order to properly control the fabricated ZnO–metal nanomaterials and correlate the developed nanostructures with the observed fluorescence enhancement and/or quenching. Additionally, clarification of the governing mechanisms is required.

Furthermore, ZnO nanostructures showed promising capabilities for SEF applications. The use of ZnO NPs as substrates for fluorescence enhancement is an alternative to overcome the shortages of metal-based substrates, such as fabrication cost and susceptibility to fluorescence quenching. Additional advantages include a high capacity for multiplex detection as well as the fact that the fluorescence enhancement does not seem to depend on the distance between fluorophores and the surface of ZnO substrates, as it happens with metal nanostructures. Nevertheless, SEF applications of ZnO and ZnO–metal nanostructures have been limited and additional studies are needed to elucidate the fluorescence enhancing capabilities and to properly clarify the responsible mechanism.
